# Multidisciplinary management of necrotizing fasciitis as a postoperative complication after mastectomy in an adult male in a low- and middle-income country

**DOI:** 10.1093/jscr/rjae412

**Published:** 2024-06-11

**Authors:** Mohammad Aadil Qamar, Alishba Rehman, Habib ur Rehman Toor

**Affiliations:** Research Department, Research X, Karachi, 75600, Pakistan; Department of Surgery, Ziauddin University, Karachi, 75600, Pakistan; Department of Surgery, Peoples University of Medical and Health Sciences for Women, Nawabshah 67840, Pakistan; Department Surgical Unit II, Peoples University of Medical and Health Sciences for Women, Nawabshah 67840, Pakistan

**Keywords:** necrotizing fasciitis, male breast cancer, Pakistan, multidisciplinary, metastasis

## Abstract

Necrotizing fasciitis, a rare, potentially life-threatening infection, often necessitates urgent medical intervention and surgical excision of the affected tissue. We present a 55-year-old male patient with a progressively enlarging lump in the left breast that was diagnosed as a breast carcinoma. Post-modified radical mastectomy, histopathological examination revealed Grade II invasive ductal carcinoma with neuroendocrine features. Due to financial constraints, the patient missed post-operative follow-ups and did not complete the prescribed radiotherapy sessions. Three months later, the patient returned with fever, swelling alongside sharp pain in the left arm and oozing blood. A clinical diagnosis of necrotizing fasciitis was made, leading to urgent surgical debridement. While the wound progressively healed, a contracture developed restricting elbow movement. An Orthopedic Review and Bone scintigraphy revealed metastasis of breast carcinoma to the sternum. This case report highlights the multi-disciplinary management required in such financially constrained rare cases in low- and middle-income countries.

## Introduction

Male breast cancer accounts for 0.5–1% of the total reported cases worldwide [1]. Apart from diabetes mellitus, trauma, etc., surgery is a risk factor in the development of necrotizing fasciitis, an extremely rare occurrence with an incidence of 0.4 per 100 000 people per year in the USA [2]. Wong et al. reported polymicrobial infection, as a common occurrence with an increased risk of mortality associated with delays more than 24 hours in the management of the condition raising the need for prompt management [3]. Though a rare infection, necrotizing fasciitis is associated with mortality rates ranging from 20 to 30% [4, 5]. Given the serious outcomes, this case report discusses the multidisciplinary approach to a case of male breast cancer, an extremely rare occurrence complicated by the post-operative complication of another extremely rare entity, necrotizing fasciitis in a financially constrained hospital setting in Pakistan.

## Case report

A 55-year-old married male, ex-smoker visited our facility reporting an increasing lump in his left breast over the past 4 years, experiencing frequent episodes of pain. A laborer by profession, he recounts a previous injury 5 years back involving a fall onto a brick, resulting in pain and minor swelling on the left side of his chest without any bleeding. Subsequently, the swelling progressed. He was successfully treated for Hepatitis C 12 years ago, and currently takes no medications with no family history of carcinoma.

Physical examination revealed moderate wasting, no signs of jaundice, apyrexia and a pale complexion. Examination of the left breast revealed a firm, immobile nodular mass positioned around the 12 o’clock mark, with ulceration of the skin around the areolar region, including retraction of the nipple and no enlarged lymph nodes. A suspicion of breast carcinoma was confirmed through a Tru-Cut biopsy.

A multidisciplinary team consisting of surgeons, oncologists and pathologists convened, resulting in the diagnosis of T2aN1Mo. A successful mastectomy was performed, raising skin flap together with inner fibrofatty to enable the mass to be accessible for removal, followed by removal of level I, II and III axillary lymph nodes and suturing. Specimen measuring 11 × 9 × 8 cm^3^ was excised and evaluated, revealing grade II invasive ductal carcinoma with neuroendocrine features. Unfortunately, due to financial and transportation limitations, the patient was unable to finish the prescribed radiotherapy treatment and follow-up appointments.

**Figure 1:**
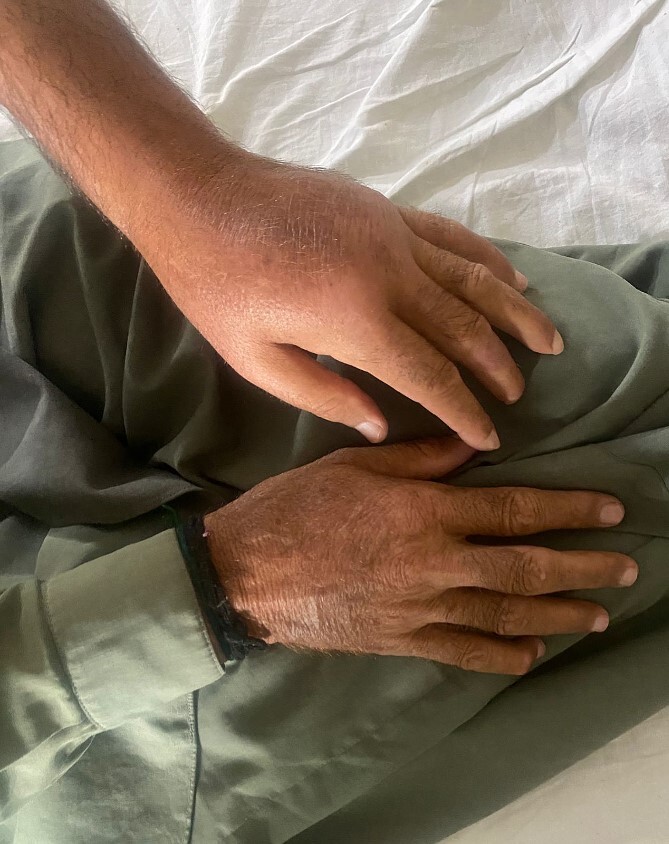
Left arm swelling compared to the right side.

Three months later, the patient revisited, presenting symptoms of swelling of the entire left arm which started from the axilla 2 weeks ago, high-grade fever for 5 days, severe pain, multiple wounds on the lateral aspect of his elbow with bloody discharge. Upon examination, there was profound edema throughout the arm, affecting all fingers, and multiple superficial wounds described as dark brown necrotic patches with bloody discharge, noted over the elbow, with signs of inflammation in the surrounding area (Fig. 1). Laboratory tests revealed elevated C-Reactive Protein (1.5 mg/dL), WBCs (18.5 × 10^9^) and HbA1c (6.4%) with decreased hemoglobin levels (12.1 g/dL).

**Figure 2:**
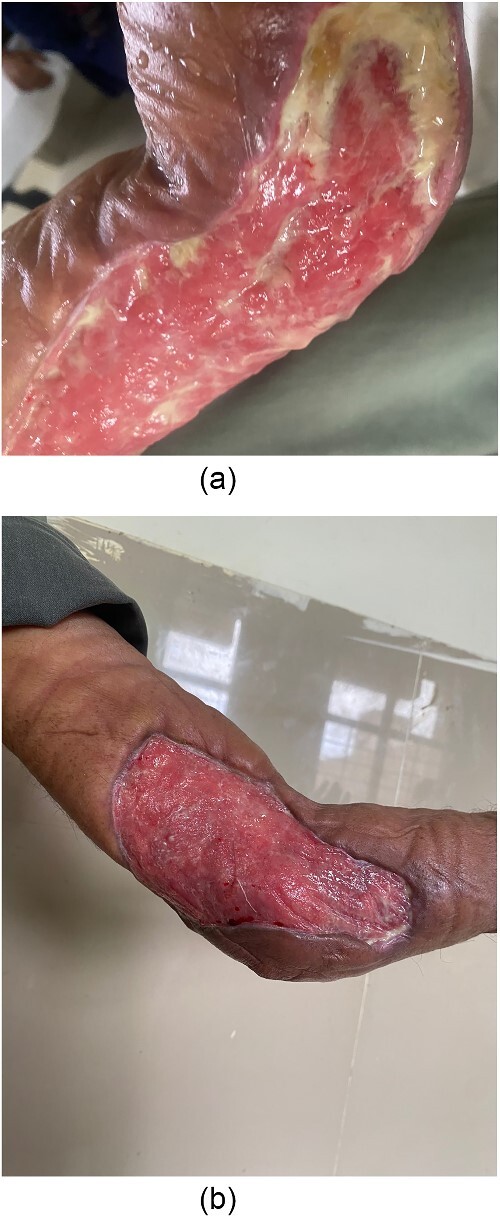
Left elbow contracture post debridement. (a) Week 2. (b) Week 6.

Upon thorough evaluation, a clinical diagnosis of cellulitis and necrotizing fasciitis was made. Consequently, the patient received IV antibiotics (amikacin 500-mg BD and Linezolid 600-mg BD) and underwent urgent debridement of the necrotic area on the second day post-admission. The patient was monitored in the hospital for two days before undergoing a second debridement session. The wound showed signs of healing, but contractures were observed. An orthopedic review and skeletal scintigraphy were conducted revealing increased radiotracer uptake on the sternum, indicating a possible metastasis. The patient started chemotherapy regimen and responded positively to it and can flex his elbow by 30° (Fig. 2).

## Discussion

Upon extensive literature review, we report that this is the first ever such case of male breast cancer providing a chance to investigate the diagnostic importance of necrotizing fasciitis while exploring treatment issues owing to financial constraints.

Necrotizing fasciitis is mainly diagnosed clinically. Following the shortcomings of the Laboratory Risk Indicator for Necrotizing Infection (LRINEC) [6], we assessed the newly developed, SIARI [Site other than lower limb, Immunosuppression, Age < 60 years, Renal impairment (creatinine >141) and Inflammatory markers (CRP ≥ 150, WBCs >25)]. Retrospective assessment of our case showed a score of 5/11, more than the cut-off of 4 for a diagnosis of necrotizing fasciitis. Based on the presenting signs and symptoms, deranged laboratory values and CT findings, we diagnosed this as a case of necrotizing fasciitis, as supported by Xin-ke Wei in their CT imaging findings [7]. Moreover, Goh et al. report swelling, pain and erythema as the leading clinical features, which are the findings consistent with ours, providing insights into a prompt diagnosis [8]. Given the incomplete laboratory work-up due to financial constraints, the SIARI system proves to be a better tool than LRINEC in a resource-scarce setting like ours.

This case was complicated owing to non-affordability issues. Though management remains same, Aziz *et al*. report a completion rate of therapy for breast cancer of 10–40% versus 40–90% in low- and middle/high-income patients, respectively [9]. As this was observed in our case, Universal Health Coverage, as per World Health Organization recommendations, needs to be implemented in Sindh, Pakistan [10]. ‘Sehat Sahulat Programme’ in Khyber Pakhtunkhwa, Pakistan, is one such example [11], providing the means to tackle complications resulting from modifiable factors such as affordability.

## Conclusion

This unique case provides valuable input on the urgent diagnosis and management of necrotizing fasciitis while highlighting the need to provide financial assistance for low-income patients.

## Data Availability

All information is mentioned in the manuscript. Further data will be made available on reasonable requests.
